# Polyphenols’ Impact on Selected Biomarkers of Brain Aging in Healthy Middle-Aged and Elderly Subjects: A Review of Clinical Trials

**DOI:** 10.3390/nu15173770

**Published:** 2023-08-29

**Authors:** Tobias Ziegler, Melina Tsiountsioura, Lisa Meixner-Goetz, Gerhard Cvirn, Manfred Lamprecht

**Affiliations:** 1 Division of Medicinal Chemistry, Otto Loewi Research Center, Medical University of Graz, 8010 Graz, Austria; tobias.ziegler@jpsi.info (T.Z.); melina.tsiountsioura@jpsi.info (M.T.); gerhard.cvirn@medunigraz.at (G.C.); 2 Juice Plus+ Science Institute, Memphis, TN 38017, USA; lisa.meixner-goetz@jpsi.info; 3 Green Beat Institute of Nutrient Research, 8010 Graz, Austria

**Keywords:** polyphenols, brain aging, BDNF, amyloid-beta, Aβ, NGF, tau, flavonoids, curcumin, resveratrol

## Abstract

With a constantly growing elderly population, incidences of neurodegenerative diseases are also rising and are expected to further increase over the next years, while costing health systems across the world trillions of dollars. Therefore, biomarkers to detect manifestations of brain aging early and interventions to slow down its pace are of great interest. In the last years, the importance of the neurotrophins brain-derived neurotrophic factor (BDNF) and nerve growth factor (NGF) in the context of cognitive function and the aging brain has increased, besides the already well-established amyloid-beta (Aβ) and tau plaques. Due to their wide range of beneficial health effects as well as their antioxidant and anti-inflammatory properties, a class of secondary plant-metabolites, the so-called polyphenols, gained increasing attention. In this review, we discuss the roles of BDNF, Aβ, NGF, and tau proteins as biomarkers of brain aging and the effect of dietary polyphenol interventions on these biomarkers, assessed via blood analysis, magnetic resonance imaging (MRI), and positron emission tomography (PET).

## 1. Introduction

During the process of aging, the human body undergoes several physiological changes, and the same holds true for the brain. In the aging brain, certain molecules, cells, vasculature, and morphology are seen to be altered, which in turn can increase susceptibility to developing diseases. For example, incidences of stroke and white matter lesions rise with age and can be accompanied by changes in levels of neurotransmitters or hormones [1]. The loss of brain volume and weight can also be a concomitant manifestation, starting at the age of 40 and accelerating further beyond the age of 70 years [2,3]. Certainly, these physical, physiological, or neurotransmitter/hormone-related changes can in turn affect cognitive function. Generally, cognitive function is assessed by investigating its subdomains such as memory, the most widely observed subdomain to be affected by aging [1]. Additionally, executive function, which is a complex construct reflecting many higher-order cognitive processes such as planning future actions, inhibiting the processing of irrelevant information, and adjusting behavior to changes, can be negatively impacted by age. The decline in these so-called executive functions has recently been stated as a characteristic of cognitive aging [4]. However, all these undesirable changes must have underlying molecular triggers, which appear to become more pronounced over the course of the aging process. Among these triggers, which ultimately have been referred to as “hallmarks of brain aging”, mitochondrial dysfunction, oxidative stress, and neuroinflammation particularly stand out [5].

While aging, chronic low-grade inflammation develops without the involvement of pathogens—a condition called “inflammaging”, which can contribute to the pathogenesis of age-related diseases. This constant state of low-grade inflammation is supposed to be sustained by several stimuli including cellular senescence-associated inflammation, external pathogens, endogenous cell debris, changes in the gut-microbiome, as well as nutrient excess or overnutrition [6,7]. Furthermore, cytokine dysregulation at an older age is reflecting the inability to fine-control systemic inflammation, characterized by a shift towards a pro-inflammatory phenotype. However, there is still no absolute clarity about the triggers of inflammaging that underline most major age-related diseases including atherosclerosis, diabetes, Alzheimer’s disease (AD), rheumatoid arthritis, cancer, and aging itself [8]. Obviously, there is a connection between systemic low-grade inflammation and neuroinflammation. In this context, neuroinflammation can be seen as an indirect consequence of systemic inflammation through inflammatory signaling to the central nervous system (CNS) [9,10]. Neuroinflammation is one of the key players associated with brain aging, since neuroinflammation was shown to be implicated in age-related neurodegenerative diseases such as AD and Parkinson’s disease [11,12]. Actual clinical studies investigating neurodegeneration are focusing on cytokines and chemokines in the blood but this approach has the limitation that systemic reactions cannot be distinguished from brain-specific ones [13]. To obtain more brain or CNS-specific information, the measurement of cytokines and chemokines in cerebrospinal fluid (CSF) appears to be a promising alternative [14,15], and interestingly, recent evidence shows that the level of CSF proinflammatory cytokines increase linearly with age [16]. The status of inflammation is likewise directly linked to oxidative stress or vice versa, since one could promote the other [17]. Oxidative stress has been described as a disorder in the prooxidative–antioxidative steady state of metabolism in favor of prooxidative processes [18]. It causes molecular damage on structures and function systems [19] as well as modifications in signal transduction [20], and can thus be seen as another key player in the context of brain aging or aging in general.

Previous research showed that the human diet has a major impact on both the prevention and the development of age-related diseases, since many plant-derived phytochemicals were found to modulate inflammatory and oxidative stress signaling [21]. Within these plant-phytochemicals, the class of polyphenols is of particular interest. Polyphenols are secondary plant metabolites that possess aromatic rings with several hydroxyl groups attached to them, while their structure can range from a simple molecule to complex polymeric and high molecular weight compounds [22,23]. Up until now, several hundred representatives are known and their content in more than 400 foods can be found summarized in a database [24]. The bioavailability of polyphenols varies a lot due to their chemical structure diversity. In foods, polyphenols are commonly present in conjugation to sugar moieties or as polymers, whereof the sugar group is called glycone and the polyphenol is known as the aglycone. In these forms, polyphenols are not bioavailable and need to be hydrolyzed by intestinal enzymes or the colonic microbiota before absorption [25,26]. Upon absorption, polyphenols exert their antioxidant effects through their radical scavenging activities. These antioxidant activities are influenced by the arrangement of functional groups as well as their nuclear structure [27] and recent evidence suggests that certain polyphenols can reform themselves after scavenging superoxide radicals, granting them properties similar to the enzyme superoxide dismutase [28]. Besides their antioxidant activities, polyphenols were also seen to modulate inflammation-associated pathways such as nuclear factor-kappa β, mitogen-activated protein kinases, Wnt/β-catenin, phosphatidylinositol 3-kinase, and protein kinase B by selective interactions with components of these pathways [29]. However, it is not only their antioxidative and anti-inflammatory properties that make dietary polyphenols so interesting in relation to brain aging but also their influence on cognitive functions. Dietary polyphenols were found to have significant beneficial effects on cognitive functions of middle-aged to elderly subjects [30] but are also thought to be capable of preserving or improving cognitive performance that precedes the onset of dementia [31]. Due to this wide range of beneficial effects related to redox activities, inflammation, and cognitive function, polyphenol interventions could be a promising approach to slow down the pace of brain aging.

Since polyphenol interventions have shown positive effects on brain aging by modulating underlying mechanisms as mentioned above, it would be of particular interest to evaluate their impact on brain-related biomarkers such as the brain-derived neurotrophic factor (BDNF), belonging to the class of neurotrophins. BDNF is well known to play an important role in the central nervous system since it can promote neuronal growth and survival and modulate synaptic plasticity. Furthermore, alterations in BDNF levels were found in neurodegenerative diseases, suggesting BDNF is a promising biomarker in most neurodegenerative conditions, which can be reliably measured in blood samples [32,33]. Another very promising and already extensively researched biomarker is the beta amyloid (Aβ) deposition in the brain, which is associated with AD and cognitive decline. Several new approaches to determine Aβ plaques have evolved, mostly based on magnetic resonance imaging (MRI) or positron emission tomography (PET) [34], but also blood levels of Aβ proteins can be a robust measure in the diagnosis of AD [35]. In addition to BDNF and Aβ, the neurotrophin NGF could also be of interest as a biomarker of brain aging since it was found to be associated with age-related diseases and abnormalities in trophic signaling which could trigger cholinergic depletion and subsequently cognitive decline [36]. Furthermore, tau proteins (the microtubule-associated proteins) were reported to play a crucial role in neurodegenerative diseases because intronic mutations in the tau-gene or age-related changes in tau function can be linked to the development of AD [37].

In this review, we focus on the interaction between different kinds of polyphenol interventions and their impact on selected biomarkers of brain aging, namely BDNF, Aβ, NGF, and tau detected in blood and via PET and/or MRI. Although the complexity of aging-linked pathways and their estimate cannot be covered by these four analytes alone, they were selected because in the current scientific literature, they appear as the most promising ones in the context of polyphenol interventions and brain aging. Furthermore, the studies selected for this review investigated polyphenol interventions in healthy, middle-aged to elderly populations.

## 2. Methodology

For this review, the PubMed and Cochrane databases were screened for clinical trials investigating the influence of polyphenol interventions on levels of BDNF, Aβ, NGF, and tau in blood as well as determined by PET and/or MRI. Therefore, the following keywords were used in combination: polyphenols, flavonoids, anthocyanins, flavanols, flavonols, phenolic acids, lignans, stilbenes, resveratrol, curcumin, and brain-derived neurotrophic factor, BDNF, nerve growth factor, NGF, amyloid-beta peptides, amyloid-beta, tau, tau protein.

**Figure 1:**
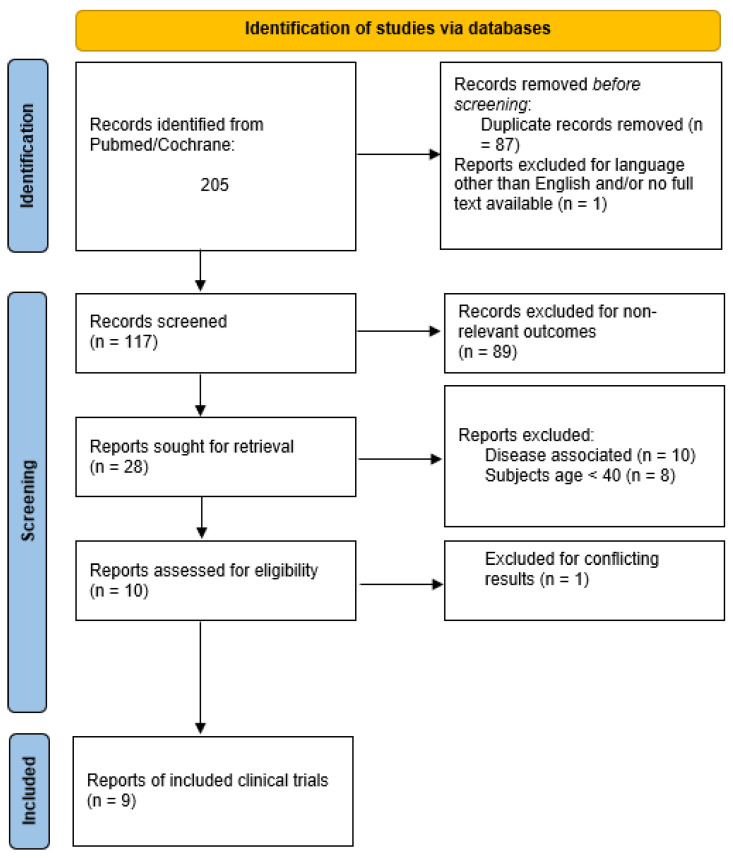
Flowchart of retrieval and selection of studies.

From all studies obtained and after exclusion of duplicates, only studies with generally healthy subjects aged 40 years and older were selected. Studies with participants younger than 40 years old and studies with subjects suffering from some kind of disease-associated condition or cognitive impairment were excluded. The study selection and retrieval process are illustrated in Figure 1.

## 3. Potential of BDNF, Aβ Peptides, NGF, and Tau Proteins as Biomarkers of Brain Aging

### 3.1. BDNF

Like other neurotrophins, BDNF is produced as a precursor protein called pro-BDNF, which is later proteolytically cleaved into mature and functional BDNF (mBDNF) [38,39]. BDNF signaling primarily occurs through its receptor tropomyosin-related kinase receptor type B (TrkB). Both BDNF and TrkB are widely distributed within the human brain, and dysfunction of BDNF-TrkB signaling is associated with neurodegenerative diseases. Upon binding of BDNF to TrkB, autophosphorylation of tyrosine residues on the cytoplasmatic domain takes place, which in turn can activate phospholipase Cγ1 (PLC-γ1), Ras-mitogen-activated protein kinase (MAPK), and phosphoinositide 3-kinases (PI3K)-protein kinase B (AKT) signaling pathways, and thus, BDNF can affect various important secondary messengers [40]. Generally, BDNF is supposed to support differentiation and to regulate the survival of neurons in the nervous system [41,42,43] and interestingly, mBDNF was found to be identical among all mammals while working with tissue specificity [44]. Another main function of BDNF in the adult brain is to regulate both excitatory and inhibitory synapses in many brain regions and it is associated with neuronal activity and synaptic plasticity [45]. Animal studies showed that blood concentrations of BDNF can directly reflect brain tissue levels of BDNF [46] and in human studies, BDNF turns out to be reliably measurable in blood [33].

It is apparent that chronological aging is directly related to brain aging, and previous research shows that BDNF levels in blood decrease as we grow older [47]. Furthermore, increasing age is associated with smaller hippocampal volume, reduced serum BDNF levels, and poorer memory performance [48]. Not only aging but also low levels of education were seen to be associated with decreased levels of BDNF, which further correlate with the severity of cognitive impairment, since AD patients were shown to express the lowest levels of BDNF [49]. However, conflicting evidence reported higher levels of plasma BDNF in AD patients as compared to subjects with mild cognitive impairment (MCI). Hence, a negative correlation between BDNF and cognitive function was found, which could be explained by the body’s attempt to counteract neurodegeneration by increasing levels of BDNF [50]. Another study suggests to not only investigate the levels of BDNF and correlate it with cognitive outcomes but also to look at the ratio of proBDNF/BDNF, since a higher ratio was seen to be linked to lower cognitive performance and the ratio could be an even better marker for cognitive function [51]. This could be related to the fact that proBDNF binds to the p75 neurotrophin receptor (p75NTR) and further signaling could lead to apoptosis [52]. In terms of methods to increase BDNF levels, moderate exercise, mental training, or exercise combined with mental training are already well established [53,54,55]. All the abovementioned facts suggest the role of BDNF as a promising biomarker of brain aging.

### 3.2. Aβ Peptides

Aβ peptides are well-recognized biomarkers associated with neurodegeneration since these peptides can form extracellular fibrils or plaques in the brain that continuously increase in size [56]. These Aβ peptides are cleaved from their much larger amyloid precursor protein (APP), an integral membrane protein expressed in many tissues but especially in synapses and neurons [57]. The APP is generally supposed to regulate synaptic formation and repair, neuronal transport, and iron export [58,59,60,61]. When APP is cleaved by β-secretases and γ-secretases, this results in a 37 to 49 amino acid long peptide—Aβ. The remaining part of APP can be internalized and further cleaved, by β-secretases and γ-secretases at multiple sites, yielding fragments of 43, 45, 46, 48, 49, and 51 amino acids, which are further cleaved into the final forms Aβ40 and Aβ42 [62,63,64]. The Aβ40 and Aβ42 peptides are the ones that mainly make up plaques and fibrils which are thought to exert neurotoxic effects but also their ratio (Aβ42/Aβ40) seems to have an impact on their toxic properties [65,66,67]. Aβ fibers or oligomers can promote the formation of new Aβ oligomers, generating greater toxic effects, and they can disrupt endosome vesicles and interfere with the transport of brain-derived neurotrophic factors. Additionally, the internalization of oligomers can trigger intracellular systemic damage, increase endoplasmic reticulum stress, mediate calcium imbalance and hence, can lead to the activation of apoptotic caspase 3 [68]. Some of the Aβ peptides (including dimers, trimers, hexamers, and 12-meres) can be imported into the mitochondria, where they bind to the inner membrane and interfere with complex I of the respiratory chain [69]. The peptides can also accumulate in lipid rafts and trigger abnormal cell signaling to reduce electrical activity in hippocampal synapses. Furthermore, Aβ peptides can form cyclic structures on the membrane to destroy its integrity, which leads to neuronal death and neurodegeneration, accompanied by inflammatory factors secreted by Aβ-activated microglia and more [68].

Nowadays, it is supposed that the Aβ pathway triggers further downstream events and actual disease models support the idea of Aβ peptides being the initiator of a pathophysiological cascade that further leads to tau misfolding and assembly, which results in neural system failure, neurodegeneration, and cognitive decline [70,71]. Recent evidence suggests that amyloid deposition in the aging brain precedes decline in cognitive functions, since, as mentioned above, amyloid plaques are the main initiator in a cascade of events, which ultimately lead to age-related cognitive decline at a later timepoint [34]. Many studies support the theory that higher loads of Aβ were seen to be associated with decline in several subdomains of cognitive function, confirming its importance as a biomarker of brain aging [72,73,74,75]. Besides MRI and PET imaging, Aβ40 and Aβ42 can be reliably measured in blood to predict current and future brain amyloidosis and progression of dementia while being consistently associated with the well-established indicators of AD [76,77]. Although there is a lot of evidence linking Aβ peptides to cross-sectional and progressive brain atrophy, cross-sectional network dysfunction, and longitudinal cognitive decline, the examination of subjects just by presence or absence of the Aβ proteins may not be informative enough to define the status of disease [78].

### 3.3. NGF and Tau Proteins

Another neurotrophin of interest in the context of brain aging is NGF. NGF can bind two classes of cell surface receptors that are expressed in the basal forebrain cholinergic nuclei (BFCN), namely, tropomyosin-related kinase a (TrkA) and p75NTR, whereof TrkA is bound with a higher affinity than p75NTR. The further signaling cascade of NGF, which is produced from neurons, involves PI3K/Akt, mitogen-activated protein kinase (MEK)/extracellular signal-regulated kinase (ERK), and PLCγ signaling pathways, which promote neuronal survival and dysfunction of the signaling pathway, and may result in impaired cytoskeleton function. Also, BFCN neurons’ survival depends on the binding of NGF, and their dysfunction is a cardinal feature of AD [79,80]. Evidence from animal studies indicates that higher levels of NGF were found to be linked with improved cognitive functions [81,82,83]. This was further supported in human studies since reduced TrkA expression and higher levels of proNGF are related to mild cognitive impairment [84]. In pathological aging, a loss of neuronal cells can be observed, whereas normal aging is accompanied by a gradual loss of cholinergic function caused by dendritic, synaptic, and axonal degeneration, as well as a decrease in trophic support. Imbalances in NGF expression and its precursor proNGF, their receptors TrkA and p75NTR, but also changes in acetylcholine release, high-affinity choline uptake, as well as muscarinic and nicotinic acetylcholine receptor expression may contribute to cholinergic dysfunction. Furthermore, Aβ peptides may trigger cholinergic dysfunction by affecting NGF signaling and mediating tau hyperphosphorylation, another biomarker of interest [85].

Tau proteins can form insoluble filaments that accumulate as neurofibrillary tangles in AD and related tauopathies. The normal function of tau is to regulate the assembly and maintenance of the cytoskeleton, but in diseased brains, tau can become modified, which causes the microtubules to disassemble and free tau protein aggregates into filaments, a process where Aβ peptides are also supposed to interact with [86]. Generally, posttranslational modifications such as hyperphosphorylation, acetylation, nitration, truncation, and others are thought to be responsible for changes in tau-structure and function and abnormal masses of intracellularly aggregated tau proteins can be toxic to neurons. Hence, tau proteins play a major role in neurodegenerative diseases [87] and also higher plasma levels of tau were reported to be associated with cognitive impairment [88].

## 4. Polyphenols’ Impact on Selected Biomarkers of Brain Aging in Middle-Aged and Elderly People: Studies’ Overview and Discussion

In this literature review we included all clinical trials on overall healthy people aged 40+, investigating the effects of any kind of polyphenol intervention on levels of BDNF, Aβ, NGF, and tau in blood or as measured by imaging techniques such as PET and/or MRI. In total, 10 clinical trials investigating the effects of polyphenol interventions on biomarkers of brain aging that fit our inclusion criteria remained after the exclusion of duplicates, studies with irrelevant outcomes, studies with subjects suffering from any kind of disease, and studies with subjects younger than 40 years old (Figure 1). Out of the 10 selected studies, one more was excluded because the reported data on BDNF measurements were conflicting, leaving nine trials to be reviewed (Table 1).

In the first clinical trial obtained from the search, an aronia berry extract was investigated for its impact on measures of cognitive function and serum levels of BDNF in a randomized double-blinded, placebo-controlled, parallel-grouped study. In this study, 101 subjects aged 40–60 years old were randomized to receive either 90 mg or 150 mg aronia extract or placebo per day, for a total duration of 24 weeks. The 90 mg aronia extract provided 16 mg of anthocyanins, whereas the 150 mg intervention provided 27 mg of anthocyanins, mostly cyanidin-3-galactoside, cyanidin-3-arabinoside, cyanidin-3-xyloside, and cyanidin-3-glucoside. Levels of BDNF in serum, measured by enzyme-linked immunosorbent assay (ELISA), did not differ between groups at any timepoint. Neither a time × treatment interaction nor an effect of time or treatment was found. Other measures of cognitive function were also not affected, except for psychomotor speed which showed improvement after the intervention with 90 mg aronia extract compared to placebo [89].

In a study by Garcia-Cordero, the effect of cocoa powder, a mix of red berries, and a combination of both on serum BDNF and nerve growth factor receptor (NGF-R) was assessed. Sixty healthy adults between 50 and 75 years old were randomly allocated to one of the intervention groups. The intervention lasted 12 weeks and measurements were performed at baseline and at 12 weeks. The cocoa powder provided around 200 mg of flavanols, whereas the mix of red berries provided approximately 100 mg of anthocyanins per day. There was no effect on serum levels of BDNF and NGF-R measured by ELISA. Remarkably, information regarding the total polyphenol intake obtained from a 3-day dietary recall revealed a positive correlation between higher polyphenol intake and higher BDNF levels, and between number of movements required to finish the cognitive tower of London test and NGF-R in men. In women, these positive correlations were found between memory, processing speed, and attention with NGF-R, indicating that higher scores in these tests were associated with higher levels of NGF [90].

Another randomized, double-blinded, parallel-armed study assessed the effect of perilla seed oil and a combination of perilla seed oil and ponkan powder on serum levels of BDNF in 49 healthy elderlies (60–85 years). The daily dose of perilla seed oil alone provided around 0.88 g of α-linolenic acid (ALA), while the combination of the oil and the ponkan powder provided the same amount of α-linolenic acid plus 2.91 mg of the flavonoid nobiletin. Participants were randomized to ingest either the perilla seed oil alone or the oil in combination with the ponkan powder for a total period of 12 months. Serum BDNF levels only increased significantly in the group ingesting the oil plus ponkan powder as compared to baseline. The mean changes in BDNF were not significantly different between the groups but BDNF levels positively correlated to Mini Mental State Examination (MMSE) scores [91].

Igwe et al. conducted a randomized, controlled, cross-over trial, with 28 healthy adults older than 55 years to elucidate whether a low anthocyanin plum nectar could improve cognition and serum BDNF levels. In this trial, the duration of each period was 8 weeks, separated by a 4-week wash-out period. Within the intervention arm, 200 mL of queen garnet plum nectar providing 7.4–10.6 mg of anthocyanins (based on intervention stability tests) was administered daily, whereas the placebo group which received a raspberry cordial with negligible anthocyanin content, matched for vitamin C levels. No significant effects on serum BDNF levels (ELISA) or cognitive function were found [92].

In a randomized controlled, double-masked, cross-over trial, 40 subjects aged 62–75 years were allocated to receive a high-flavanol (HF) cocoa drink or a low-flavanol (LF) cocoa drink for 28 days, of which the HF provided 494 mg of flavanols per day and the LF provided 23 mg of total flavanols per day. The wash-out period between the two different interventions was 4 weeks and serum BDNF levels were measured using ELISA. After a total duration of 12 weeks, the HF intervention was associated with a statistically significant increase in serum BDNF levels and global cognitive function relative to the LF intervention. In combination with data from another trial of the same group, the authors found a positive correlation between serum BDNF levels and age in subjects younger than 65 years. On the contrary, for subjects older than 65 years, a significant negative correlation between BDNF and age was observed. Additionally, when BDNF data were split into tertiles of age (35–50; 51–65; 66–90), the findings indicated that subjects aged 51–65 had significantly higher levels of serum BDNF than subjects aged 35–50. Furthermore, the age group of 66–90 years had significantly lower levels of BDNF than the age group of 35–50 years. These data indicate that BDNF levels increase until the age of 65 and then start to decrease. Furthermore, increased BDNF levels were associated with improved global cognition [93].

Baba et al. investigated the effect of supplementation with 336.4 g of catechins per day for 12 weeks on cognitive outcomes as well as serum BDNF and Aβ peptides in subjects reporting self-assessed cognitive decline but still classified as cognitively normal. The results of the study show that there were no significant differences between placebo and catechin-treated subjects in terms of amyloid-β1-40 or amyloid-β1-42 or the Aβ1-40/Aβ1-42 ratio. Also, for the amyloid-β precursor protein α (sAPPα), the amyloid-β precursor protein (APP)770, and BDNF levels, no significant differences were found, although the catechin intake was associated with beneficial effects on cognition [94].

In a placebo-controlled, randomized, double-blinded, and parallel-grouped study, Nakamura et al. studied the effect of a barley tea intervention on Aβ42/Aβ40 and overall cognitive function. Eighty participants aged 60–80 years, with age-related cognitive decline but still categorized as cognitively normal, were randomized to receive 500 mL of barley tea per day, providing 110 mg of quercetin, or a similar placebo beverage containing no quercetin for 40 weeks. Plasma levels of Aβ42/Aβ40 decreased in both groups after 40 weeks but without significant differences between the tea and placebo group. No significant differences in cognitive function were found between groups but improvements in cerebral blood flow were observed following intervention with quercetin-rich barley tea [95].

In a randomized, double-blinded, placebo-controlled, two-grouped parallel study, 40 middle-aged to elderly subjects (50–90 years) with no dementia were allocated to either placebo or highly bioavailable curcumin intervention. Subjects in the intervention group were asked to ingest 180 mg of curcumin per day for 18 months to test its effects on cognitive outcomes as well as Aβ and tau plaques. The molecule 2-(1-ethylidene) malononitrile (FDDNP) was used to measure Aβ and tau plaques or tangles in selected brain regions via PET. After 18 months of intervention with curcumin, mean FDDNP binding in the amygdala declined significantly when compared to baseline. However, no significant differences between groups were seen. Hypothalamic FDDNP binding was significantly different between groups after 18 months, whereof in the intervention group, no significant change to baseline was found but the binding levels in the placebo group significantly increased over time. There was no significant difference in FDDNP binding between groups at baseline. These findings suggest beneficial effects of curcumin on Aβ and tau accumulation in the amygdala and the hypothalamus [96].

In another placebo-controlled study, the effect of curcumin on plasma levels of Aβ measured by ELISA was investigated. In this study, 38 healthy males and postmenopausal females were assigned to receive a curcuma powder, providing 80 mg of curcumin per day, or a placebo for 4 weeks. The results of the study showed a decrease in plasma levels of Aβ after the ingestion of curcumin compared with baseline levels but not after placebo ingestion [97].

In summary, six of the included studies measured BDNF levels in serum, of which only one included data about NGF-R/NGF, all measured by ELISA. BDNF levels were affected by the intervention in only two out of the six studies, while no impact was found on NGF-R/NGF levels. The intervention periods of these studies lasted between 12 and 24 weeks and only flavonoid interventions were chosen which makes the studies comparable in some way. Inconsistency in BDNF results could be due to the fact that the measurement of BDNF in serum was reported to be reliable, but only if study populations are big enough—a group size of 60 would be needed to detect a 20% change [33]. In the studies included in our review, sample sizes ranged between 28 and 101 subjects. Furthermore, poor reproducibility of the results obtained from commercially available ELISA kits has been reported. In a study by Polacchini et al., five commercially available ELISA kits and one multiplexing assay were compared by using the sera of 40 healthy adults to overcome the limitation of variations in sample collection. The sample recovery in all samples was very good and within a comparable range but the inter-assay variations ranged from 5 to 20%. In addition, some kits can react with both mature and proBDNF while others only bind mature BDNF, which could also explain the inconsistency across studies [98].

In terms of Aβ and tau proteins, four studies were included according to our criteria. Of the three studies measuring Aβ peptides in plasma or serum via ELISA, one accounted for several different Aβ peptides in serum, one measured the plasma Aβ42/Aβ40 ratio, and another one determined Aβ in plasma. The fourth study was assessing Aβ and tau protein aggregates via PET, and with the method used, a distinction between Aβ and tau was not made. Two of the four studies assessing Aβ peptides in blood reported no significant effects of the intervention, whereas significant changes were seen in PET results and in one study measuring plasma Aβ levels. Also, the reliability of the measurements needs to be taken into account, since it was reported that immunoassays for Aβ performed not as well as mass spectrometry-based measurements [99]. In addition, plasma Aβ assessments might not distinguish between Aβ-positive or negative scans in PET but still, the plasma Aβ42 and Aβ42/Aβ40 ratio was associated with Aβ-PET status [100]. The two studies that reported not significant changes used flavonoid interventions, while in the studies showing significant changes, curcumin was ingested, suggesting the idea that curcumin could have more impact on Aβ levels as measured by PET or in blood. However, two studies in total are by far not enough to determine a clear picture. Additionally, the studies supplementing flavonoids investigated their effects in a population that already reported mild age-related cognitive decline, which is also a factor that needs to be considered. The intervention periods of the studies varied between 4 and 18 months and subject numbers ranged from 38 to 80. The use of both curcumin and flavonoid supplementations renders the studies hardly comparable. Nevertheless, decreased plasma levels of Aβ and prevention of its further accumulation in the brain were reported. In addition, one study reported that intervention with the flavonoid genistein prevented Aβ-uptake into the dentate gyrus over a period of 12 months measured by FDDNP-PET [101].

## 5. Conclusions and Future Perspectives

From the studies reviewed, no clear conclusions can be drawn regarding the relationship between dietary polyphenol interventions and their impact on biomarkers such as BDNF, Aβ, NGF, and tau. The literature on this topic is scarce, and although promising results were reported in some studies, no clear trend across studies was seen, especially when considering only the healthy middle-aged to elderly population. Additionally, the fact that different methods were used to assess the biomarkers of interest makes it even harder to obtain an overview of the actual relationship between polyphenol consumption and the selected biomarkers of brain aging. Not only is it difficult to compare results obtained by different methods such as PET and measurements in plasma or serum via ELISA, but also the discrepancies observed between different commercial ELISA kits makes it almost impossible to clearly conclude a relationship. Consequently, researchers should find a consensus to harmonize the methodologies used for the measurements of biomarkers such as BDNF, Aβ, NGF, and tau. Finally, the four selected biomarkers alone cannot cover all complex brain aging-linked processes associated with plant-based nutrition sufficiently. This topic of research is still in its early stages and therefore, there is a strong need for more clinical studies to explore the effects of polyphenol interventions on markers of brain aging in healthy elderly people.

## Figures and Tables

**Table 1:** Summary of clinical trials.

Reference	Subjects	Study Design	Intervention	Main Results
Ahles 2020 [89]	Healthy adults, age 40–60	Randomized, placebo-controlled, double-blinded, parallel-grouped study	Aronia extract in capsules providing 16–27 mg of anthocyanins/day for 24 weeks or placebo	No effects on serum BDNF
Garcia-Cordero 2021 [90]	Healthy adults, age 50–75	Randomized, double-blinded, parallel-grouped study	Red berry powder (100 mg anthocyanins/day), cocoa powder (200 mg flavanols/day) or both combined and dissolved in water for 12 weeks	No effects on serum BDNF and NGF-R, but positive correlation between total polyphenol intake and BDNF levels
Hashimoto 2022 [91]	Healthy elderlies, age 60–85	Randomized, double-blinded, parallel-grouped study	Encapsulated perilla seed oil (0.88 g ALA/day) alone or in combination with ponkan powder (2.91 mg nobiletin/day) for 12 months	Serum BDNF levels increased in oil + powder group over time
Igwe 2020 [92]	Healthy adults, age > 55	Randomized, controlled, cross-over trial	Plum nectar providing 7.4–10.6 mg anthocyanins/day for 8 weeks compared to raspberry cordial	No effects on serum levels of BDNF
Neshatdoust 2016 [93]	Healthy adults, age 62–75	Randomized, controlled, double-masked, cross-over trial	High flavanol (494 mg flavanols/day) or low flavanol (23 mg flavanols/day) cocoa drink for 28 days	High flavanol intervention was associated with increased serum BDNF levels compared to low flavanol intervention
Baba 2020 [94]	Healthy adults and elderlies, age 50–69 with mild cognitive decline (MMSE score ≥ 24)	Randomized, placebo-controlled, double-blinded, parallel-grouped study	Capsules providing 336.4 g/day of catechins in capsules for 12 weeks or placebo	No significant differences between placebo and catechin group for serum Aβ1-40, Aβ1-42, Aβ1-40/Aβ1-42 ratio, Aβ precursor protein α (sAPPα), Aβ precursor protein (APP)770, and serum BDNF levels
Nakamura 2022 [95]	Healthy elderlies, age 60–80 with mild cognitive decline (MMSE score ≥ 24)	Randomized, placebo-controlled, double-blinded, parallel-grouped study	Barley tea beverage providing 110 mg quercetin/day for 40 weeks or placebo beverage	Plasma levels of Aβ42/Aβ40 decreased in both groups but change was only significant in placebo
Small 2018 [96]	Healthy middle-aged and elderlies, age 50–90	Randomized, placebo-controlled double-blinded, parallel-grouped study	180 mg of encapsulated curcumin/day for 18 months or placebo	Significant reduction of Aβ and tau plaques in the amygdala (PET measurement) compared to baseline, no change of hypothalamic Aβ and tau plaques in curcumin group but significantly increased in placebo
Di Silvestro 2012 [97]	Healthy middle-aged, age 40–60 years	Placebo-controlled, parallel-grouped study	80 mg of encapsulated curcumin/day for 4 weeks or placebo	Plasma Aβ levels decreased in curcumin group but not in placebo group

## Data Availability

No data were used for the research described in this article.
